# Analysis of Biomechanical and Biochemical Markers of Rat *Muscle Soleus* Fatigue Processes Development during Long-Term Use of C_60_ Fullerene and N-Acetylcysteine

**DOI:** 10.3390/nano12091552

**Published:** 2022-05-04

**Authors:** Dmytro Nozdrenko, Svitlana Prylutska, Kateryna Bogutska, Vsevolod Cherepanov, Anton Senenko, Oksana Vygovska, Sergii Khrapatyi, Uwe Ritter, Yuriy Prylutskyy, Jacek Piosik

**Affiliations:** 1 Department of Biophysics and Medical Informatics, ESC “Institute of Biology and Medicine”, Taras Shevchenko National University of Kyiv, 01601 Kyiv, Ukraine; ddd@univ.kiev.ua (D.N.); bogutska_ki@knu.ua (K.B.); 2 Department of Physiology, Plant Biochemistry and Bioenergetics, Faculty of Plant Protection, Biotechnology and Ecology, National University of Life and Environmental Science of Ukraine, 03041 Kyiv, Ukraine; psvit_1977@ukr.net; 3 Department of Physical Electronics, Institute of Physics, NAS of Ukraine, 03028 Kyiv, Ukraine; vvch2000@ukr.net (V.C.); senenkoanton@gmail.com (A.S.); 4 Department of Pediatric Infectious Diseases, Bogomolets National Medical University of Kyiv, 01601 Kyiv, Ukraine; ovvigovskaya@gmail.com; 5 Department of Computational Mathematics and Computer Modeling, Interregional Academy of Personnel Management, 03039 Kyiv, Ukraine; khrapatiysv@ukr.net; 6 Institute of Chemistry and Biotechnology, Technical University of Ilmenau, 98693 Ilmenau, Germany; uwe.ritter@tu-ilmenau.de; 7 Intercollegiate Faculty of Biotechnology, University of Gdansk and Medical University of Gdansk, 80-307 Gdansk, Poland

**Keywords:** *muscle soleus*, muscle fatigue, C_60_ fullerene, N-acetylcysteine, biomechanical parameters of muscle contraction, biochemical parameters of blood

## Abstract

The development of an effective therapy aimed at restoring muscle dysfunctions in clinical and sports medicine, as well as optimizing working activity in general remains an urgent task today. Modern nanobiotechnologies are able to solve many clinical and social health problems, in particular, they offer new therapeutic approaches using biocompatible and bioavailable nanostructures with specific bioactivity. Therefore, the nanosized carbon molecule, C_60_ fullerene, as a powerful antioxidant, is very attractive. In this study, a comparative analysis of the dynamic of *muscle soleus* fatigue processes in rats was conducted using 50 Hz stimulation for 5 s with three consistent pools after intraperitoneal administration of the following antioxidants: C_60_ fullerene (a daily dose of 1 mg/kg one hour prior to the start of the experiment) and N-acetylcysteine (NAC; a daily dose of 150 mg/kg one hour prior to the start of the experiment) during five days. Changes in the integrated power of muscle contraction, levels of the maximum and minimum contraction force generation, time of reduction of the contraction force by 50% of its maximum value, achievement of the maximum force response, and delay of the beginning of a single contraction force response were analyzed as biomechanical markers of fatigue processes. Levels of creatinine, creatine phosphokinase, lactate, and lactate dehydrogenase, as well as pro- and antioxidant balance (thiobarbituric acid reactive substances, hydrogen peroxide, reduced glutathione, and catalase activity) in the blood of rats were analyzed as biochemical markers of fatigue processes. The obtained data indicate that applied therapeutic drugs have the most significant effects on the 2nd and especially the 3rd stimulation pools. Thus, the application of C_60_ fullerene has a (50–80)% stronger effect on the resumption of muscle biomechanics after the beginning of fatigue than NAC on the first day of the experiment. There is a clear trend toward a positive change in all studied biochemical parameters by about (12–15)% after therapeutic administration of NAC and by (20–25)% after using C_60_ fullerene throughout the experiment. These findings demonstrate the promise of using C_60_ fullerenes as potential therapeutic nanoagents that can reduce or adjust the pathological conditions of the muscular system that occur during fatigue processes in skeletal muscles.

## 1. Introduction

One of the most important characteristics of the human muscular system, along with the magnitude of force generated by skeletal muscles, is their ability to maintain the level of effort generation that was given by the central nervous system (CNS) over a period of time. Muscle fatigue occurs when contraction-inducing factors continue to flow to the muscle with constant intensity, and the level of strength generated by the muscle itself gradually decreases. Currently, there is no single separate mechanism of muscle fatigue development, and there is a set of mechanisms at different system levels [1]: in particular, disorders of the CNS [2], dysfunction of peripheral nerves and neuromuscular junctions [3], physiological reversible changes directly in the skeletal muscles [4,5] that perform the work.

Accumulated data evidence that disruption of the “excitation–contraction” coupling is most likely localized in muscle fibers; this explains the fatigue-induced decrease in maximum strength in humans, while central (neural) fatigue plays a greater role in the event of an inability to continue sustained low-intensity contraction [6]. Based on data from intact single muscle fibers, fatigue-induced impairment of “excitation–contraction” includes: a decrease in the number of active cross-bridges due to the reduction of Ca^2+^ ions release; a decrease of myofilaments sensitivity to Ca^2+^; reduction of the force generated by each active cross-bridge.

Calcium ions play a critical role as initiators and preservatives of the cross-bridging cycle in the formation of skeletal muscle strength. The authors of [7] presented a new chemo-mechanical model for analyzing the role of Ca^2+^ in muscle fatigue, as well as predicting muscle fatigue. It is assumed that even minor disturbance of myocytes integrity, imbalance of “Ca^2+^—muscle pathology” can play an important role in the rate and degree of development of fatigue processes [7,8].

Deceleration of the contractile properties of skeletal muscles is one of the characteristic signs of fatigue [9]. There are three factors that contribute to the loss of power by mammalian muscles at physiological temperatures: a decrease in isometric strength, which mainly indicates a decrease in the number of active cross-bridges, a slowdown in the maximum speed of unloaded shortening, and an increase in the curvature of the force-velocity ratio [9]. This last change is the main reason for the loss of muscle power during the development of pathologies associated with changes in the integrity of the myocyte membrane complex [10].

The dynamic of the contractile component is determined by subtle mechanisms of interaction between motor neuron pools that occur in muscle via activated motor neuron and activation of the interaction between actin and myosin filaments. Under isometric conditions, the analysis of the recorded force developed by the muscle during frequency-modulated stimulation of its nerve is a qualitative indicator of the level of myopathic pathological processes [11]. Rapid processes of excitation of the contractile apparatus in the process of long-term activation of the muscle fiber usually undergo a slow and steady modification, which may partly be associated with phosphorylation of myosin light chains located in the neck of the bridge. A slower process of dephosphorylation under conditions of prolonged continuous activation of the muscle fiber causes stable phosphorylation of myosin, which, apparently, increases the mobility of the bridges or changes their orientation [12]. Analysis of the amplitude-velocity changes in the force response (biomechanical markers of the contractile process) of the activated muscle gives the possibility to assess the level of influence of the developing pathology on these processes [13]. These processes play an important role in precise positioning movements of the hand and fingers: even minor disturbances in the control system of these movements lead to very serious physiological problems [14].

Literature data indicate that free radicals are an important pathogenic factor in the process of muscle fatigue [15,16]. They include initiation of lipid peroxidation (LPO), direct inhibition of mitochondrial respiratory chain enzymes and ATPase activity, inactivation of glyceraldehyde-3-phosphate dehydrogenase and membrane sodium channels, etc. [17,18]. One of the mechanisms by which free radicals cause tissue damage is the interaction of the hydroxyl radical with the hydrogen atoms of the methyl groups of polyunsaturated fatty acids. This process initiates POL, which, in turn, leads to the increase in permeability of cell membranes [19].

The ability of the biocompatible C_60_ fullerenes and their derivatives to inactivate the reactive oxygen species (ROS) was first demonstrated by Krustic et al. [20]. It has been established that pristine C_60_ fullerenes have a dose-dependent protective effect against oxidative-mediated muscle trauma [21,22]. Moreover, C_60_ fullerene protects the rat’s liver from ROS [23,24]. Given the accumulated data about the powerful antioxidant properties of C_60_ fullerenes [25], the purpose of this work was to conduct a comparative analysis of changes in biomechanical and biochemical markers of *muscle soleus* fatigue processes development in rats during long-term therapeutic use of C_60_ fullerene and the well-known antioxidant N-acetylcysteine (NAC) [26].

## 2. Materials and Methods

### 2.1. Preparation of C_60_FAS

For the preparation of C_60_ fullerene aqueous colloid solution (C_60_FAS) at a maximum concentration of 0.15 mg/mL, we used a saturated solution of pristine C_60_ fullerene (purity > 99.96%) in toluene with a C_60_ molecule concentration corresponding to maximum solubility near 2.9 mg/mL, and the same amount of distilled water in an open beaker. The two phases formed were treated in an ultrasonic bath. The procedure was continued until the toluene had completely evaporated and the water phase became yellow colored. Filtration of the aqueous solution allowed to separate the product from undissolved C_60_ fullerenes [27,28]. The prepared C_60_FAS is stable within 12–18 months at temperature +4 °C.

### 2.2. AFM and STM Analysis

The atomic force microscopy (AFM) and scanning tunneling microscopy (STM) were performed to determine the size of C_60_ fullerene particles (their aggregates) in an aqueous solution. Measurements were done with the “Solver Pro M” system (NT-MDT, Moscow, Russia). A drop of investigated solution was transferred to the atomic-smooth substrate to deposit layers. Measurements were carried out after complete evaporation of the solvent. For AFM studies, a freshly broken surface of mica (SPI supplies, V-1 grade) was used as a substrate. Measurements were carried out in a semicontact (tapping) mode with AFM probes of the RTPESPA150 (Bruker, 6 N/m, 150 kHz) type. STM studies were performed with the Au (111) surface obtained after annealing substrates of Au/mica (Phasis, Geneva, Switzerland) in a gas burner flame (propane-butane). The typical tunneling current and voltage values were 0.027–0.1 nA and 0.1–1 V, respectively.

### 2.3. Animals

Male Wistar rats (170 ± 12 g, 2-month-old) were bred and housed in standard temperature conditions (21–23 °C), a lighting (12/12 h light-dark cycle), at humidity (30–35)%. All animals had unlimited access to chow and tap water. The study was carried out in strict accordance with the European convention for the protection of vertebrate animals used for experimental and other scientific purposes (Strasbourg, 1986) and was approved by the Bioethical Committee of the ESC “Institute of Biology and Medicine” of Taras Shevchenko National University of Kyiv, Ukraine.

Four experimental groups of animals (*n* = 7 in each group) were studied: after C_60_FAS and NAC administration, which were compared with the control group (“fatigue”, no drug administration) and the intact group.

Based on our previously obtained data [11,29], the research protocol involved intraperitoneal injection of C_60_FAS and NAC at a daily dose of 1 and 150 mg/kg, respectively, one hour before the experiment for 5 days.

It is important to note that water-soluble C_60_ fullerenes at low concentrations did not manifest any toxic effects as to normal cells [30,31]. Moreover, the selected dose of C_60_FAS in our experiments is significantly lower than the LD_50_ value, which was 600 mg/kg body weight when administered orally to rats [23] and 721 mg/kg when administered intraperitoneally to mice [31].

### 2.4. Biomechanical and Biochemical Analysis

The object of the study was the rat *muscle soleus*. In preliminary preparation for the experiment, anesthesia was performed by intraabdominal injection of nembutal (40 mg/kg). Standard preparation included cannulation (*a. carotis communis sinistra*) for pressure measurement and laminectomy at the lumbar spinal cord level. *Muscle soleus* was released from surrounding tissues, and their tendon parts were connected to force measurement sensors in the distal part. To prepare for modulated efferent stimulation, the ventral roots in the respective segments were transected directly at their exit points from the spinal cord.

The dynamic properties of muscle contraction were studied under conditions of muscle activation using the method of modulated efferent stimulation [32]. Fatigue was induced by successive stimulation impulses with a frequency of 50 Hz and a duration of 5 s each, without a relaxation period between them. The sum of such stimulation signals was 500 s, followed by 5 min of relaxation. The number of stimulation pools was three. The external load on the muscle was controlled using a system of mechanostimulators. Changes in contraction force were measured by strain gauges. During the analysis of the results, we used the following quantitative parameters: integrated muscle power, levels of maximum and minimum strength generation of contraction, time of reduction of the contraction force by 50% of its maximum value, achievement of the maximum force response, and delay of the beginning of single contraction force response.

The levels of creatinine, creatine phosphokinase (CPK), lactate (LA), lactate dehydrogenase (LDH), thiobarbituric acid reactive substances (TBARS), hydrogen peroxide (H_2_O_2_), reduced glutathione (GSH), and catalase (CAT) activity as markers of muscle injury [33] were determined in the blood plasma of experimental animals using clinical diagnostic equipment—a haemoanalyzer [21].

### 2.5. Statistical Analysis

Statistical processing of the measurement results was performed by methods of variational statistics using the software Origin 9.4. Each of the experimental force curves obtained in the work is the result of averaging 10 similar experiments. At least three repetitions were performed for each biochemical measurement. Data are expressed as means ±SEM for each group. Differences from experimental groups were indicated by one-way ANOVA described by Bonferroni’s multiple comparison test. Values of *p* < 0.05 were considered significant.

## 3. Results and Discussion

### 3.1. Characterization of C_60_FAS

**Figure 1:**
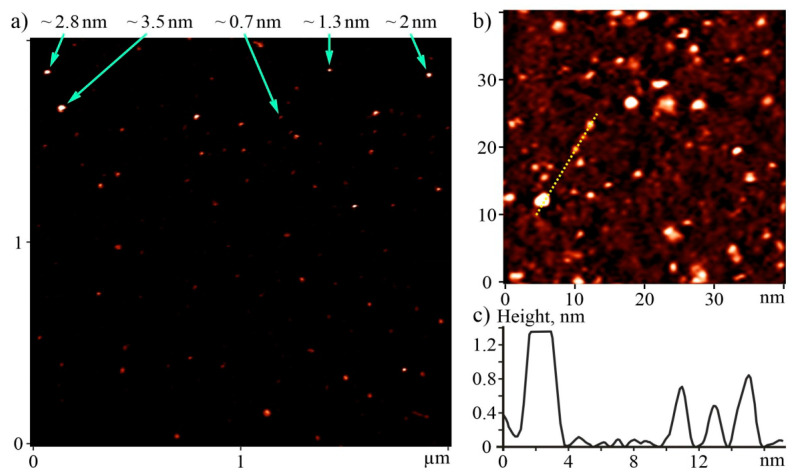
AFM image of C_60_ fullerene layer deposited from C_60_FAS on a mica substrate (**a**). The values near the arrows indicate the height of the nanoobjects including single C_60_ fullerene (~0.7 nm); STM image of C_60_ fullerene layer deposited from C_60_FAS on an Au(111) substrate (**b**). *Z*-profile along the dashed line marked on the STM image (**c**).

The AFM images of the C_60_ fullerene layers show casually placed point-shaped objects up to 10 nm high, mostly 0.7–3.5 nm (Figure 1a). The height of the smallest objects 0.7 ± 0.2 nm) agrees well with the molecular diameter of C_60_ fullerene, which allowed them to be identified as individual molecules. Larger objects correspond to C_60_ fullerene bulk clusters. We analyzed the statistics of the distribution of objects by height according to data on 100 randomly selected molecules or clusters within a certain area of the surface. Depending on the height, they were included in one of the size groups from 0.7 ± 0.2 nm to 3.5 ± 0.2 nm, which corresponds to one to five diameters of the C_60_ molecule. According to statistics, the relative number of single C_60_ molecules was 60%, clusters with a height of ~1.3 nm—28%, and clusters of other size groups (~2.0 nm, ~2.8 nm, and ~3.5 nm)—4% each. The clusters had symmetrical bell-shaped *Z*-profiles with sharp maxima in the middle. This indicates that they were formed in solution long before application to the substrate.

The STM method allowed us to more accurately determine the lateral sizes of objects. On STM images, the majority of objects also had a point-like shape and a height of 0.75 ± 0.15 nm or 1.3 ± 0.2 nm (Figure 1b,c), which is consistent with the value of one or two molecular diameters of C_60_ fullerene. Among them, the relative part of the former was ~80%, and the width of their profiles at half height was 1.5–1.8 nm (Figure 1c). For STM, the minimum possible tip radius is equal to the radius of its closest atom to the surface, to which the width of the tunnel gap (~0.2 nm) should be added. Our STM probe was made of an alloy of platinum and iridium, whose atomic radii are ~0.18 nm. Thus, C_60_ molecules, which can be modeled as spheres with a diameter of ~0.7 nm, will have a lateral size of at least 0.7 + 2 × (0.2 + 0.18) = ~1.5 nm on STM images. This proves that the objects visible in the images with a height of ~0.75 nm and a diameter of 1.5–1.8 nm are individual molecules. Objects of greater height correspond to clusters of C_60_ fullerene. Analysis of the profiles showed that their shape is close to spherical. The existence of such aggregates in aqueous solutions was predicted earlier theoretically [34].

Thus, the data of both methods (AFM and STM) indicate that C_60_ fullerene was in the non-aggregated or low-aggregated state in the studied solutions. The isolated arrangement of C_60_ molecules (clusters) is explained by the existence of electrostatic repulsion forces between them, namely they demonstrated a high negative surface charge (zeta potential value was −25.3 mV at room temperature [31]), indicating a very low tendency for aggregation in aqueous solution (i.e., a high solute stabilization).

### 3.2. Biomechanical Analysis

**Figure 2:**
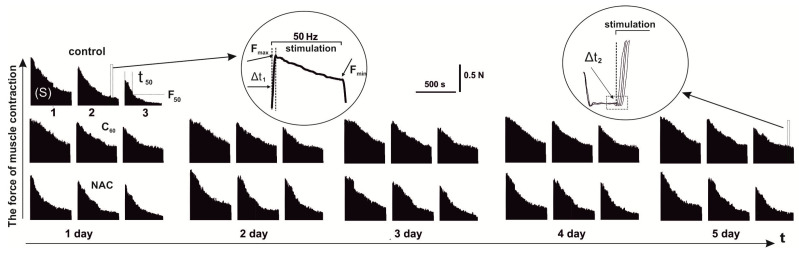
Recording the contractile force of rat *muscle soleus* after application of 50 Hz stimulation for 5 s in three consecutive pools (1,2,3) for 500 s each with 5 min relaxation between them: native muscle (control) and muscle after C_60_FAS (C_60_) and NAC administration during 1, 2, 3, 4, and 5 days. S—integrated muscle power (calculated area under the power curve); F_max_ and F_min_—maximum and minimum strength of a single contraction; Δt_1_—the time to reach the maximum force of a single muscle contraction; Δt_2_—time of muscle force response beginning; t_50_—time of decreasing the muscle contraction force by 50% (F_50_) of the maximum value in the contraction pool.

Despite the emergence of new experimental approaches to the analysis of neuromuscular regulation processes at the microlevel, traditional electrophysiological models using neuromuscular preparation in vivo are of paramount importance [4]. These studies should be carried out not only for the purpose of a more accurate quantitative analysis of the pathologies of muscle dynamics but also for a detailed study of the aggregation of the central processes involved in the regulation of muscle contraction. Figure 2 shows the change in contractile strength of rat *muscle soleus* after application of 50 Hz stimulation for 5 s in three successive pools for 500 s each with 5 min of relaxation between them after C_60_FAS and NAC administration during 5 days.

Several basic biomechanical parameters were taken into consideration for analyzing the miotic response of the studied muscle [35]. The presence of changes in each of them indicates dysfunction of a certain link in the “excitation-response of the muscle preparation” chain.

**Figure 3:**
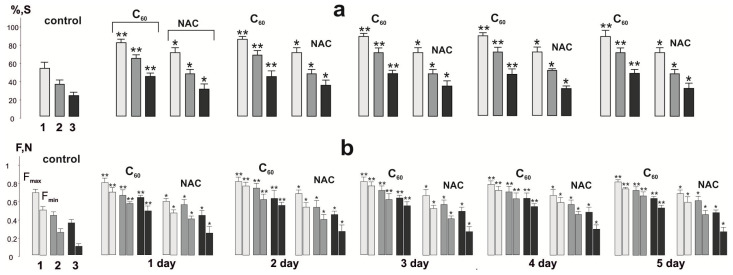
The integrated power of the rat *muscle soleus* (S, presented as a percentage of the maximum values) (**a**) and the peak values of its contraction force (F, N) (**b**) after applying 50 Hz stimulation for 5 s in three consecutive pools (1,2, 3) for 500 s each with 5 min relaxation between them: native muscle (control); muscle after administration of C_60_FAS (C_60_) and NAC for 1, 2, 3, 4, and 5 days. F_max_ and F_min_ are the maximum and minimum forces of a single muscle contraction. * *p* < 0.05 compared to control; ** *p* < 0.05 compared to values in the NAC group.

*Change in the integrated power of muscle contraction.* Integrated power, as the calculated area under the power curve (Figure 2 and Figure 3), is an indicator of the overall performance of the muscle with the applied stimulation pools [22]. The analysis of this parameter makes it possible to evaluate the kinetics of the formation of muscle fatigue in the “force–external load” equilibrium system.

The change in the integrated power of rat *muscle soleus* (Figure 2 and Figure 3) showed its significant decrease already after 1st stimulation pool, which amounted to 58 ± 4%. After the relaxation period, the integrated power progressively decreased at the 2nd and 3rd stimulation pool, which was 39 ± 2% and 24 ± 3%, respectively.

The use of NAC increased the value of this indicator to 77 ± 5%, 50 ± 5%, and 31 ± 3% at 1, 2, and 3 stimulation pools, respectively. Thus, the therapeutic effect of NAC was 32%, 28%, and 25% at 1st, 2nd, and 3rd stimulation pools, respectively (compared to control). Using of NAC injections during the next 4 days did not show significant changes in integrated power.

Injection of C_60_FAS led to an increase in the level of this parameter to 82 ± 4%, 66 ± 5%, and 42 ± 3% at 1, 2, and 3 stimulation pools, respectively. Thus, the protective effect after the first injection of C_60_FAS was 41 ± 3%, 69 ± 6%, and 75 ± 3% at 1st, 2nd, and 3rd stimulation pools, respectively (compared to control). There is a progressive increase in the therapeutic effect of C_60_FAS throughout the experiment. So, on the 5th day of C_60_FAS therapy, the indicators of integrated power were 89 ± 3%, 77 ± 3%, and 50 ± 3% at the 1st, 2nd, and 3rd stimulation pool, respectively, which corresponds to an increase in its effect by 8%, 16%, and 19% compared to a single injection. It should be noted that a further increase in the duration of C_60_FAS application did not lead to statistically significant changes in muscle biomechanics and, therefore, is not presented in this work. In our opinion, this may be due to the establishment of the maximum possible equilibrium concentration of C_60_ fullerene in the active muscle on the 5th day of its use.

*Changing the generation levels of maximum (F_max_) and minimum (F_min_) contraction force.* The F_max_ marker is an indicator of the general dysfunction of the muscular system, namely, an indicator of a decrease in the maximum possible force response during the development of fatigue (Figure 2 and Figure 3). A change in this parameter can be associated with both the development of fatigue processes in the neural component and the miotic components of the studied pathology [36]. Its dysfunction, in our opinion, can also be due to a violation of the integrity of the signals that generate motor neurons into the synaptic current, which leads to a violation of the summation of transmembrane currents in accordance with the internal membrane properties. All this affects the pathological transformation of the sequence of action potentials that trigger muscle contraction, causing a maximum force response.

The F_min_ marker is an indicator of the maximum pathological changes during the development of fatigue processes in each successive contractile act (Figure 2 and Figure 3). While performing simple single-joint movements, this marker is the main indicator of muscle dysfunction, the phenomenological analysis of which makes it possible to establish the presence of causal relationships between the level of decrease in muscle biomechanical activity, the main mechanical parameters of movements, and the level of development of the pathological process. If there is a difference between the maximum and minimum force response of an active muscle with a constant frequency stimulation signal, there are serious difficulties in correcting the control of muscle strength by the CNS, which, in turn, makes it difficult to correct the precise positioning of the joints, which is observed during the development of muscle fatigue.

The analysis of the obtained mechanograms showed that the maximum force indicators of contraction were 0.70 ± 0.08 N, 0.42 ± 0.05 N, and 0.39 ± 0.05 N at 1st, 2nd, and 3rd stimulation pools, respectively, in the control measurements (Figure 3). Thus, the decrease in maximum strength was 40% and 55% of the initial values at the 2nd and 3rd contraction pools, respectively.

The use of NAC did not significantly change the maximum contraction force of any of the three studied stimulation pools during a five-day application.

Injections of C_60_FAS resulted in a change in maximum contraction force to 0.81 ± 0.10 N, 0.65 ± 0.05 N, and 0.63 ± 0.05 N at 1st, 2nd, and 3rd stimulation pools, respectively, after the first injection. Thus, the therapeutic effect of C_60_FAS was 15%, 54%, and 63% at 1st, 2nd, and 3rd contraction pools, respectively. The therapeutic effect of C_60_FAS for five days led to the following change in the above indicator: 0.82 ± 0.10 N, 0.75 ± 0.05 N, and 0.64 ± 0.05 N at 1st, 2nd, and 3rd pools stimulation, respectively. As noted, there is practically no difference in the maximum contraction force after using C_60_FAS at all stimulation pools, in contrast to an almost 50% decrease in this indicator in the control values. Thus, this therapy supports the stabilization of the mechanokinetics of the contractile process during prolonged activations of the studied muscle.

The minimum indicators of the force response of the studied muscle showed its decrease to 0.50 ± 0.04 N, 0.21 ± 0.03 N, and 0.14 ± 0.01 N at 1st, 2nd, and 3rd stimulation pools, respectively, in control measurements (Figure 3). It should be noted that at the 3rd contraction pool, the decrease in the minimum force was five times less than the initial value, which indicates the presence of very significant fatigue processes in the muscle due to an insufficient relaxation period.

Injections of NAC increased the minimum strength values, which were 0.57 ± 0.05 N, 0.41 ± 0.05 N, and 0.27 ± 0.02 N at 1st, 2nd, and 3rd stimulation pools, respectively. Thus, the application of NAC significantly corrected the minimum force of contraction only at the 2nd and 3rd pool of contractions. At the same time, the five-day use of this drug did not significantly increase the indicators of the minimum contraction force.

C_60_FAS injections significantly changed the mechanokinetics of the contractile process: the minimum force was 0.72 ± 0.05 N, 0.59 ± 0.06 N, and 0.51 ± 0.05 N at 1st, 2nd, and 3rd stimulation pools, respectively, after the first injection. On the 5th day of C_60_FAS administration, these indicators were 0.79 ± 0.06 N, 0.65 ± 0.05 N, and 0.59 ± 0.05 N at 1st, 2nd, and 3rd pools, respectively. Thus, the therapeutic effect of C_60_FAS was 58%, 209%, and 421% after a single application and increased by (13–16)% after a five-day administration. Thus, the maximum effects of C_60_FAS application are observed at the 3rd contraction pool, which, in turn, shows the strongest violations of contraction biomechanics during the development of skeletal muscle fatigue.

**Figure 4:**
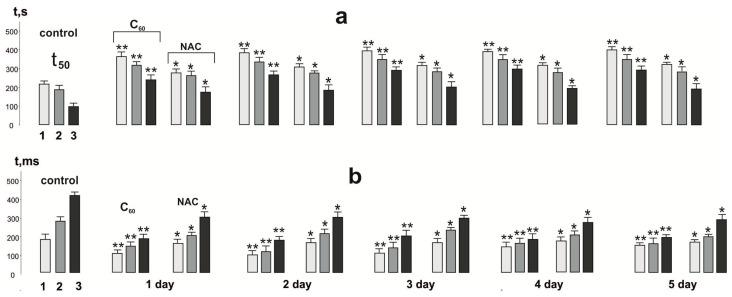
Time of contraction force decreasing in *muscle soleus* by 50% of its maximum value (t_50_) in each of the three pools of contractions (1,2,3) (**a**) and the time of reaching the maximum force of a single contraction of the muscle (**b**) after using 50 Hz stimulation for 5 s in three consecutive pools lasting 500 s each with 5 min relaxation between them: native muscle (control); muscle after injection of C_60_FAS (C_60_) and NAC for 1, 2, 3, 4 and 5 days. * *p* < 0.05 compared to control; ** *p* < 0.05 compared to NAC group.

*Change in the time of contraction force decrease in muscle soleus by 50% of its maximum value (t_50_).* We analyzed the time of decrease in rat *muscle soleus* contraction force by 50% of its maximum value in each of the three stimulation pools, which made it possible to assess the development of fatigue in different time ranges (Figure 4). In fact, this parameter is a marker of the “activation” of adaptive mechanisms that prevent the onset of progressive fatigue.

In the control, this indicator was 210 ± 12 s, 190 ± 8 s, and 104 ± 6 s at 1st, 2nd, and 3rd pools, respectively. According to the presented data, the most significant violations (more than 200%) occur in the 3rd pool of the contractile process.

After NAC application, these values were 282 ± 10 s, 275 ± 7 s, and 180 ± 7 s, which is a significant indicator of the therapeutic effect of this drug on the studied marker (33%, 44%, and 81%, respectively). It should be noted that NAC produces the maximum effect at the 3rd stimulation pool and, as in previous studies, does not change its effect during five days of use.

The use of C_60_FAS changed the time of decrease in the contraction force of rat *muscle soleus* by 50% of its maximum value, which was 380 ± 14 s, 310 ± 11 s, and 260 ± 16 s at 1st, 2nd, and 3rd pools, respectively, after the first injection. In percentage terms, these indexes were 180%, 163%, and 260%, respectively. Use of C_60_FAS during five days increased these indicators by (14–18)%.

*Change in the time of reaching the maximum force response.* High-frequency stimulation of peripheral afferents that form monosynaptic contacts with a motor neuron causes an effective summation of successive action potentials and a stable depolarization of the cell membrane [37]. In this case, the impulse frequency is determined by the average level of membrane depolarization and increases with a rise in stimulation frequency. With the development of fatigue processes in the muscle, a characteristic adaptive decrease in the time of stimulus conduction through the nervous tissue and an increase in the latent period preceding the onset of muscle force generation become noticeable. A change in this indicator is a characteristic marker of the presence of pathological processes in the neuromuscular preparation associated with the triggering of the beginning of the interaction of the myocyte actin-myosin complex [37]. Under the conditions of this experiment, the time to achieve the maximum force response during the development of fatigue processes in the control was 190 ± 12 ms, 293 ± 17 ms, and 419 ± 27 ms at 1st, 2nd, and 3rd stimulation pools, respectively (Figure 4).

After NAC injection, these values were 181 ± 15 ms, 208 ± 12 ms, and 310 ± 11 ms, respectively. Thus, the therapeutic effect was 5%, 30%, and 29%, respectively. As well as the analogous markers, this indicator showed significant differences only at the 2nd and 3rd stimulation pool and did not change after five days of NAC use.

The use of C_60_FAS changed this indicator by 108 ± 12 s, 152 ± 17 s, and 191 ± 12 s at 1st, 2nd, and 3rd pools, respectively, after the first injection. In percentage terms, these values were 75%, 92%, and 119%, respectively. It is important to note that the five-day use of C_60_FAS did not significantly change this indicator, which may be due to the maximum saturation of the active muscle with fullerene C_60_ already on the first day of its use to achieve the maximum strength response and establish the optimal concentration range.

*Change in time delays the force response beginning of a single contraction.* The time parameters of the conduction of stimulation pools along the axon do not remain constant either with a change in the intensity of stimulation or in the relaxation time. The study of changes in time delays in the conduction of impulses with an increase in the number of stimuli allowed us to assess the level of pathological processes in the neuromuscular preparation during long-term reactions of the muscular system. In a detailed analysis of the development of pathological processes associated with the development of skeletal muscle fatigue, it is necessary to use long-term stimulation pools that cause long-term transsynaptic activation of motor neurons [38]. At the same time, a characteristic adaptive decrease in the time of stimulus conduction through the nervous tissue becomes noticeable, induced by the development of fatigue processes. The change in this indicator is a characteristic marker of the presence of pathological processes in the neuromuscular preparation after using stimulation signals close to physiological parameters.

**Figure 5:**
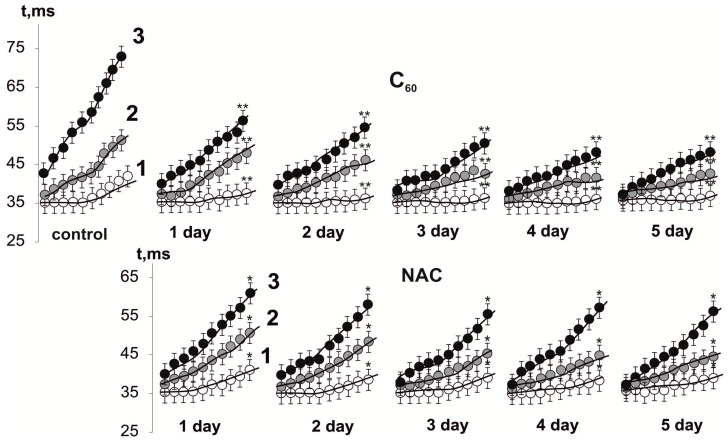
Time delay of force response beginning of 10 single contractions in rat *muscle soleus* after applying 50 Hz stimulation for 5 s in three consecutive pools (1, 2, 3) of 500 s each with 5 min of relaxation between them: native muscle (control); muscle after injection of C_60_FAS (C_60_) and NAC for 1, 2, 3, 4, and 5 days. * *p* < 0.05 compared to control; ** *p* < 0.05 compared to NAC group.

Figure 5 shows mechanograms of 10 contractions that were taken sequentially at equal time intervals from each stimulation pool using 50 Hz stimulation for 5 s in three consecutive pools, showing changes in the time of the beginning of rat *muscle soleus* force response. In the control, the value of the time delay for the beginning of the force response was (31–38) ± 3 ms for 1–10 single contractions at the 1st stimulation pool and progressively increased to (36–45) ± 2 ms, (42–74) ± 4 ms at the 2nd and 3rd pools, respectively. Thus, the increase in the time delay of force response beginning at the last contraction of the 3rd stimulation pool was about 100%.

These values were (35–34) ± 1 ms, (36–43) ± 2 ms, and (39–59) ± 4 ms, respectively, at 1st, 2nd, and 3rd stimulation pools after NAC injection. Thus, the therapeutic effect was about 25% only in the 3rd stimulation pool. Five-day therapy with this drug did not show significant differences.

The use of C_60_FAS also changed this indicator: (35–36) ± 2 ms, (37–42) ± 5 ms, and (39–48) ± 5 ms, respectively, which amounted to about 54% of the therapeutic effect at the 3rd stimulation pool. Five-day use of C_60_FAS increased this indicator by another (8–12)%.

### 3.3. Biochemical Analysis

**Figure 6:**
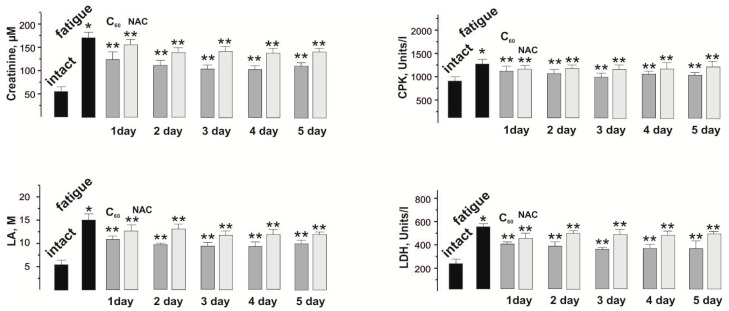
Biochemical indicators of the fatigue processes development in rat *muscle soleus*: the levels of creatinine, CPK, LA, and LDH in the blood after application of three-component stimulation and administration of C_60_FAS (C_60_) and NAC for 1, 2, 3, 4, and 5 days. * *p* < 0.05 relative to intact group; ** *p* < 0.05 relative to the fatigue group (without drugs).

The change in the chemical composition of the blood during the development of fatigue processes is a reflection of the biochemical shifts that occur in the skeletal muscle during active work [39]. Therefore, the analysis of the biochemical composition of the blood provides both a direct assessment of the biochemical changes that occur in the muscle during prolonged contraction and the ability to evaluate the therapeutic effect of the drug used on pathological processes. Selected for the study biochemical indicators such as the levels of creatinine, CPK, LA, and LDH are markers of physiological disorders of muscle tissue due to the development of fatigue and related dysfunctions of the muscular apparatus (Figure 6).

A change in the level of creatinine, a product formed in muscles during the destruction of intramuscular structures while prolonged active work, makes it possible to assess the level of damage to myocytes during prolonged contractions. This indicator increased from 50 ± 2 µM in the intact group to 169 ± 5 µM after the application of three-component stimulation.

The use of NAC reduced this indicator to 152 ± 2 µM after a single injection and reduced its value by no more than 3% during five days of therapy with this drug.

The use of C_60_FAS reduced this indicator to 122 ± 2 µM after a single injection and reduced its value by no more than 7% during a five-day therapy with this drug. A significant decrease in the creatinine fraction (27% of therapeutic effect), in our opinion, is caused by the protective effect of C_60_ fullerenes, its molecules protect the membranes of skeletal muscle cells from nonspecific free radical damage by actively absorbing free radicals.

CPK is an enzyme found in high concentration in skeletal muscle. With mechanical damage that occurs during prolonged muscle activity, this enzyme is released from the cells with a further increase in its level in the blood. The increase in the CPK fraction in the blood during the experiment from 960 ± 13 Units/L in the intact group to 1280 ± 22 Units/L is the result of a cascade physiological violation of the integrity of the walls of myocytes, which increases with active long-term relaxation-free contraction.

Injections of NAC and C_60_FAS led to a decrease in this enzyme to 1280 ± 24 Units/L and 1092 ± 27 Units/L, respectively, and a slight increase in the therapeutic effect of C_60_FAS (8%) was observed only by the 5th day of the experiment.

In an active muscle, most metabolic processes occur under anaerobic conditions, as a result of which the muscle uses a large number of mitochondrial enzymes and, as a result, there is an accumulation of a large amount of LA, which does not have time to be oxidized during prolonged muscle stimulation [39]. An increase in the level of lactic acid in an active muscle indicates that the amount of its entry into the cell exceeds the amount of its oxidation and output. In the intact group, the LA level was 5.0 ± 0.4 M. After fatigue initiation, its value increased to 16 ± 1 M.

Injections of NAC and C_60_FAS reduced lactate levels to 13 ± 1 M and 11 ± 1 M, respectively. Five-day C_60_FAS therapy reduced the LA level to 9.0 ± 0.5 M. Thus, C_60_FAS therapy led to an increase in LA oxidation by almost 40% compared to the control (“fatigue”), which turned out to be more effective than NAC 35%.

The level of change in LDH, an enzyme that catalyzes the oxidation of lactic acid, made it possible to assess the general state of muscle performance after the beginning of fatigue. The change in the level of this enzyme from 210 ± 11 Units/L in the intact group to 540 ± 12 Units/L after induced fatigue is evidence of the development of significant muscle dysfunctions associated with an excess of fatigue pathogens.

Injections of NAC reduced the content of LDH to 490 ± 11 Units/L and its value did not change significantly during the five days of the experiment.

Injection of C_60_FAS reduced the level of LDH to 400 ± 11 Units/L after the first administration and to 380 ± 11 Units/L on the 5th day of its use.

**Figure 7:**
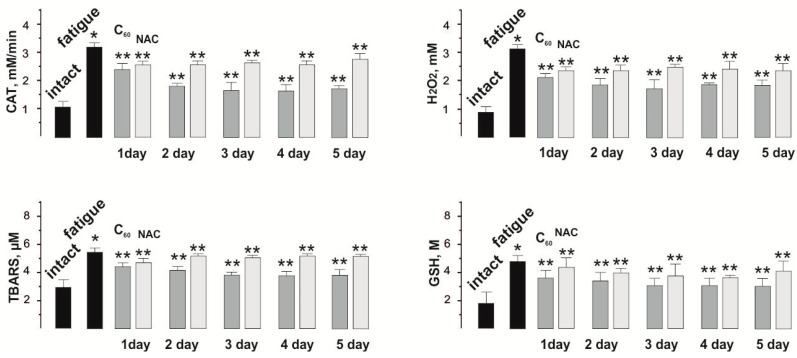
Indicators of pro- and antioxidant balance (CAT, H_2_O_2_, TBARS, and GSH) in the blood of rats after application of three-component stimulation and administration of C_60_FAS (C_60_) and NAC for 1, 2, 3, 4, and 5 days. * *p* < 0.05 relative to intact group; ** *p* < 0.05 relative to the fatigue group (without drugs).

Inflammatory cascade processes that occur immediately after the initiation of fatigue in the skeletal muscle are a source of ROS and contribute to the intensification of LPO processes [7]. This prevents the muscles from adequately performing work and significantly increases the length of the recovery period. The data obtained clearly demonstrate an increased level of markers of peroxidation and oxidative stress (CAT, H_2_O_2_, TBARS, and GSH) after the beginning of muscle fatigue and their decrease due to the applied therapy (Figure 7).

Thus, CAT activity increased from 0.9 ± 0.1 mM/min in the intact group to 3.2 ± 0.1 mM/min after the development of muscle fatigue.

Injections of NAC and C_60_FAS reduced CAT activity to 2.7 ± 0.1 mM/min and 2.5 ± 0.1 mM/min, respectively, during the course of the experiment.

The level of H_2_O_2_ was 3.1 ± 0.2 mM during the development of fatigue (0.8 ± 0.1 mM in the intact group) and 2.4 ± 0.2 mM and 2.1 ± 0.2 mM after the administration of NAC and C_60_FAS, respectively, during the experiment.

The change in TBARS level was 5.8 ± 0.2 μM during the development of fatigue (2.5 ± 0.3 μM in the intact group) and 4.3 ± 0.1 μM and 4.1 ± 0.4 μM after administration of NAC and C_60_FAS, respectively, during the experiment.

The content of GSH was 5.7 ± 0.6 M during the development of fatigue (1.9 ± 0.2 M in the intact group) and 4.2 ± 0.4 M and 3.8 ± 0.4 after administration of NAC and C_60_FAS, respectively, during the experiment.

Thus, the above in vivo results demonstrate a positive cumulative effect on the resumption of the contractile function of skeletal muscles with the therapeutic use of biocompatible water-soluble C_60_ fullerenes. This opens up broad prospects for their use in a complex of rehabilitation procedures aimed at restoring motor activity, in clinical and sports medicine, as well as for preliminary therapeutic procedures before work, associated with extreme physical exertion. However, this requires further clinical trials (dose-effect).

## 4. Conclusions

Thus, the obtained data indicate that the applied therapeutic drugs have the most significant effects on the 2nd and especially the 3rd pool of skeletal muscle stimulation. The use of C_60_FAS on the first day by (50–80)% has a stronger effect on the resumption of muscle biomechanics after the beginning of fatigue than NAC, and its five-day use additionally increases the therapeutic effect by (12–15)% for all studied biomechanical markers, except for the time of reaching maximum force response. There is also a positive therapeutic trend towards a decrease in all described biochemical parameters by about (12–15)% after administration of NAC and by (20–25)% after using C_60_FAS. Five-day use of NAC did not significantly change the studied parameters, while long-term use of C_60_FAS increased the therapeutic effect by about (7–9)%. This indicates the presence of a more powerful compensatory activation of the endogenous antioxidant system by C_60_ fullerene in response to prolonged muscle stimulation.

In summarizing, C_60_ fullerene can influence the activity of endogenous antioxidants, preventing dysfunction in an active muscle and, thus, maintaining it within the physiological norm throughout the entire process of its contraction. This provides the potential possibility of using C_60_FAS as a therapeutic agent capable of reducing and correcting the pathological conditions of the muscular system that occur during fatigue processes in the skeletal muscles.

## Data Availability

The data presented in this study are available on request from the corresponding author.
